# Sarcoma epidemiology and cancer-related hospitalisation in Western Australia from 1982 to 2016: a descriptive study using linked administrative data

**DOI:** 10.1186/s12885-020-07103-w

**Published:** 2020-07-06

**Authors:** Cameron M. Wright, Georgia Halkett, Richard Carey Smith, Rachael Moorin

**Affiliations:** 1 Health Economics and Data Analytics, School of Public Health, Faculty of Health Sciences, Curtin University, GPO Box U1987, Perth, Western Australia 6845 Australia; 2 School of Medicine, College of Health & Medicine, University of Tasmania, Churchill Avenue, Hobart, Tasmania 7005 Australia; 3 School of Nursing, Midwifery and Paramedicine, Faculty of Health Sciences, Curtin University, Perth, Western Australia 6102 Australia; 4 Department of Orthopaedic Surgery, Sir Charles Gardner Hospital, Hospital Ave, Nedlands, Western Australia 6009 Australia; 5 Centre for Health Services Research, School of Population and Global Health, Faculty of Medicine, Dentistry and Health Sciences, University of Western Australia, 35 Stirling Highway, Crawley, Western Australia 6009 Australia

**Keywords:** Sarcoma, Epidemiology, Incidence, Prevalence, Survival

## Abstract

**Background:**

Sarcomas are a heterogeneous group of malignancies arising from mesenchymal cells. Epidemiological studies on sarcoma from Australia are lacking, as previous studies have focused on a sarcoma type (e.g. soft tissue) or anatomical sites.

**Methods:**

Linked cancer registry, hospital morbidity and death registration data were available for Western Australia (WA) from 1982 to 2016. All new sarcoma cases among WA residents were included to estimate incidence, prevalence, relative survival and cancer-related hospitalisation, using the Information Network on Rare Cancers (RARECARENet) definitions. To provide a reference point, comparisons were made with female breast, colorectal, prostate and lung cancers.

**Results:**

For 2012–16, the combined sarcoma crude annual incidence was 7.3 per 100,000, with the majority of these soft tissue sarcoma (STS, incidence of 5.9 per 100,000). The age-standardised incidence and prevalence of STS increased over time, while bone sarcoma remained more stable. Five-year relative survival for the period 2012–16 for STS was 65% for STS (higher than lung cancer, but lower than prostate, female breast and colorectal cancers), while five-year relative survival was 71% for bone sarcoma. Cancer-related hospitalisations cost an estimated $(Australian) 29.1 million over the study period.

**Conclusions:**

STS incidence has increased over time in WA, with an increasing proportion of people diagnosed aged ≥65 years. The analysis of health service use showed sarcoma had a lower mean episode of cancer-related hospitalisation compared to the reference cancers in 2016, but the mean cost per prevalent person was higher for sarcoma than for female breast, colorectal and prostate cancers.

## Background

Sarcomas are a heterogeneous group of malignancies arising from mesenchymal cells. The incidence of soft tissue sarcoma (STS) is proportionately much higher than malignant osseous and chondromatous neoplasms (hereafter referred to as ‘bone sarcoma’) [1].

In Australia there are some published studies on sarcoma epidemiology. Woods et al. [2] reported an annual incidence of 1.59 per 100,000 for head and neck sarcoma from 1982 to 2009. The Cancer Council Queensland included sarcoma in their overview of childhood cancer in Australia [3], finding an increased incidence of osteosarcoma by 1.1% over the period 1983 to 2005. Work using the former Western Australian (WA) Bone Tumour Registry, now incorporated into the WA Cancer Registry (WACR) found from 1972 to 1996 there were 263 cases of primary malignant bone tumour reported in WA [4]. Authors of an Australian study of dermal sarcoma reported a mean annual incidence of 2.09 per 100,000 for cases between 1982 and 2009 [5]. A recent study focusing on STS found the age-standardised incidence increased in Australia from 4.7 to 5.87 per 100,000 from 1982 to 2009 [6].

In WA there is a lack of contemporary information about the burden of sarcoma. Basic descriptive epidemiology – incidence, prevalence, survival – is essential to guide efforts to manage and plan resources for management of patients with sarcoma. Furthermore, description of the health service utilisation can provide insight into patterns of care for those diagnosed with sarcoma. These data can also be used to support a recent Australian Government program which aims to increase clinical trial activity in rare cancers and rare diseases [7].

The aim of this study was therefore to determine the burden of sarcoma in terms of incidence, prevalence, relative survival, use and costs of hospital services in WA between 1982 and 2016, inclusive. As this information in isolation lacks a reference point, comparison was made to four common cancers: female breast, colorectal, prostate and lung cancers.

## Methods

The reporting of this population-based retrospective cohort study is based on the Reporting of studies Conducted using Observational Routinely-collected health Data (RECORD) statement [8]. The study was approved by the WA Department of Health Human Research Ethics Committee (2012/42), which exempted the study from obtaining individual patient consent.

### Data sources and linkage

The data sources analysed were: (i) the WACR; (ii) the hospital morbidity data collection (HMDC, from 1998), and; (iii) death registrations. These datasets are routinely linked by the WA Data Linkage Branch [9]. At June 2016 WA had a population of ~ 2.56 million (10.6% of the national population of ~ 24.2 million [10]).

### Description of participants

WACR data from 1 January 1982 to 31 December 2016 were used. All new, invasive malignant cases among WA residents were included in the incidence and prevalence analyses, including cancers of unknown primary site. Multiple primary cancers were included, with the WACR following the International Association of Cancer Registry (IARC) rules for multiple primary cancers [11]. Multiple primary cancers are separate records in the same individual, but not a metastasis from an initial primary. Usually multiple primaries are in separate topographical (anatomical) sites, but histologically different malignancies in the same site would be considered as two separate primaries (e.g. a breast carcinoma and a Phyllodes tumour of the breast would both be recorded). Kaposi’s sarcoma is only counted once per individual using the IARC rules, even if identified in multiple body sites at different times. For the survival analyses, records with an unknown age at diagnosis, death certificate only diagnoses, an age > 115 years at censoring, a date of death prior to the diagnostic date, or with no survival time (i.e. diagnostic date equal to date of death) were excluded [12]. For the hospital analysis, the chronologically first sarcoma from the group used in the survival analysis was linked to subsequent hospitalisations presentations (i.e. only hospitalisations occurring post-diagnosis were included).

### Selection of cancer types

Sarcoma was selected using the latest definitions and three tier hierarchy reported by the Information Network on Rare Cancers (RARECARENet) [13] (Additional file 1). Tier 1 refers to soft tissue sarcoma, bone sarcoma, gastro-intestinal stromal tumour (GIST) and Kaposi sarcoma. For this study, sarcoma was considered the sum of the tier 1 entities. This is consistent with the approach by Gatta et al. [14], though these authors classified Kaposi sarcoma as skin cancers and non-cutaneous melanoma. The tier 2 allocation separates soft tissue sarcoma anatomically and bone sarcoma into its origin in bone, cartilage etc. Tier 3 considers histology. Histological classification systems have evolved considerably over the study period, with the description of new entities and reclassification of others. For the purposes of the study, diagnoses recorded at the time were retained, albeit if some have now been modified or replaced e.g. malignant fibrous histiocytoma.

This topographical and histological inclusion is different to the allocation of sarcoma published on the Cancer Australia website [1], but allowed for a more detailed description of sarcoma epidemiology. The reference cancer types were female breast (International Classification of Disease (ICD)-10 code C50), colorectal (ICD-10 codes C18-C20, C218), prostate (ICD-10 code C61), and lung (ICD-10 codes C33, C34).

### Outcomes

Study outcomes were; (i) incidence; (ii) corrected prevalence; (iii) relative survival, and; (iv) annual total and rate of hospitalisation and associated costs.

### Statistical analysis

All analyses were conducted using Stata SE Version 15 (College Station, Texas).

#### Descriptive statistics

Descriptive statistics were generated stratified by the following diagnostic periods: 1982–87; 1988–93; 1994–99; 2000–05; 2006–11, and; 2012–16. Differences in categorical variables between periods were assessed statistically using the Pearson’s chi-squared test or Fisher’s Exact test (the latter for small cell sizes), while continuous variables were assessed using the Kruskal-Wallis test.

#### Incidence

Age-standardised incidence per 100,000 was calculated using the WA mid-year populations published by the Australian Bureau of Statistics (ABS), stratified into 5-year age groups [10]. The European Standard Population (2013) was used as a reference for age-standardisation, to allow comparison of incidence between periods to incidence reported by RARECARENet [15]. Crude incidence by broad diagnostic age group and sex was also reported for 2016.

#### Prevalence

Prevalence was calculated at 30 June for each diagnostic year by summing incident cases prior to this date among individuals who had not died. Because the prevalent period was 34.5 years (i.e. 1 January 1982 to 30 June 2016), it was assumed that the prevalence in 2016 was accurate. However, for previous years, there was less follow-up and thus a higher chance that people previously diagnosed with sarcoma and still alive were diagnosed before 1982 and therefore not counted among the prevalent population. The approach taken with an earlier analysis of WA data by Maxwell et al. [16] was adopted to correct for this. First, the number of individuals who would be prevalent in 2016, had the start of the study period been 1 January 2016, was calculated. This was then repeated working backwards by 1 year (i.e. 1 January 2015, 1 January 2014 and so on). This generated a proportion of the ‘actual’ prevalent population at mid-2016 which could then be used to generate a ‘correction factor’ to multiply by the apparent prevalence for each year according to the equation:

$$ \mathrm{CP}={\mathrm{P}}_{\mathrm{x}}/\left({\mathrm{P}}_{2016\mathrm{Yyears}}/{\mathrm{P}}_{2016,34.5\ \mathrm{years}}\right) $$

Where CP = corrected prevalence, P = prevalence, X = the year to be corrected, and Y = the number of years of look-back data available for year X. For example, if there were 1000 cases prevalent in mid-2016, but that number would have been 50 if the start of the study period had been 2016 instead of 1982, this would yield a ‘correction factor’ of 50/1000 (=0.05) for mid-1982, where there was only 6 months of diagnostic data available. If the measured prevalence in mid-1982 was 10, the corrected prevalence would then be 200 (10 divided by 0.05). As GIST was first recognised as a diagnostic entity during the study period, this was not reported separately in the prevalence analysis.

#### Relative survival

Relative survival was estimated using the Ederer II method and a period approach for 2012–2016, using the – strs – user-written command [17, 18]. The relative survival approach compared survival of the group with sarcoma to that of the general WA population. Relative survival is one of several cancer survival measures (see Baade et al. [19]). It was selected for this study because the measure is reported for sarcoma by RARECARENet [20] and Cancer Australia [1]. Single year-age and sex-specific death rates for WA published by the ABS were used [21]. For ages where there were no data, the mean of the previous and subsequent years was used. Individual cancer records ‘entered’ at 1 January 2012 and were followed to the first of all-cause death (failure) or 31 December 2016. The date of death according to the mortality registry was used, unless there was date uncertainty or the date of death was missing, in which case the date of death recorded in the cancer registry was used.

#### Health service use

Linked hospital admission records were considered cancer-related if they contained a cancer principal diagnosis, chemotherapy or radiotherapy procedure codes [16] (Additional file 1). The total number of episodes and mean episodes per corrected prevalent person, along with the total cost and mean cost per prevalent person, was reported by year of admission. Inter-hospital transfers were considered as a single episode. The cost of each episode of care was assigned based on average price weight for each Australian Refined Diagnosis Related Group (AR-DRG) code specific to the date of separation of each hospital record [22]. Cost values were reported in Australian dollars ($), adjusted to March 2019 using consumer price indices [23].

## Results

### Descriptive statistics

In total 4512 records met the inclusion criteria. Of these, 523 (12%) were excluded on the basis of not being malignant (191 (4%) benign, 315 (7%) uncertain if malignant or benign and 8 (0.2%) primary malignant, but not new). A further 11 (0.2%) were for non-WA residents and 1 (0.02%) was a duplicate record. This left 3989 sarcoma records (3 records fulfilled more than one exclusion criteria) for the analysis of incidence and prevalence (Table 1). A further 23 were excluded for the survival analysis, and a further 15 excluded for the health service utilisation analysis (*n* = 3951 included). Incident records comprised 54% males and 88% were diagnosed at ≥25 years (Table 1). Seventy-six percent of cases were STS (*n* = 3024), with 595 bone sarcomas (15%), 223 GISTs (6%) and 147 (4%) Kaposi sarcomas. The proportion of STS among the ≥65 age group increased over time (*p* = 0.03). Major notable differences between the broad sarcoma types were a tendency toward younger age at diagnosis for bone sarcoma (median age at diagnosis 38 years, compared to STS at 58 years) and a lower all-cause death (44.4%) and higher median follow-up for this group (5.4 years). Exceptions to the broad trends in age distribution and sex were a high proportion (79%) of those with STS of the uterus and STS of the peritoneum and retroperitoneum (60%) being aged 25–64 years, a higher proportion female than male among those with STS of the genitourinary tract (52%) and peritoneum and retroperitoneum (55%), and 50.5% of osseous Ewing’s sarcoma being diagnosed aged 0–14 years.

**Table 1:** Descriptive statistics for sarcoma in Western Australia, 1982–2016

	1982–87	1988–93	1994–99	2000–05	2006–11	2012–16	***p***-value^**a**^	All
**Soft tissue sarcoma**								
**Number**	304	365	450	539	632	734		3024
**Sex**								
Male (%)	140 (46.1)	185 (50.7)	245 (54.4)	280 (51.9)	337 (53.3)	395 (53.8)	0.226	1582 (52.3)
Female (%)	164 (53.9)	180 (49.3)	205 (45.6)	259 (48.1)	295 (46.7)	339 (46.2)		1442 (47.7)
**Diagnostic age group**								
0–14 years (%)	18 (5.9)	17 (4.7)	23 (5.1)	19 (3.5)	32 (5.1)	23 (3.1)	0.030	132 (4.4)
15–24 years (%)	18 (5.9)	25 (6.8)	27 (6)	25 (4.6)	27 (4.3)	24 (3.3)		146 (4.8)
25–64 years (%)	179 (58.9)	191 (52.3)	225 (50)	310 (57.5)	346 (54.7)	400 (54.5)		1651 (54.6)
≥ 65 years (%)	89 (29.3)	132 (36.2)	175 (38.9)	185 (34.3)	227 (35.9)	287 (39.1)		1095 (36.2)
**Median age (IQR)**	54 (39–68)	57 (37–73)	59 (42–72)	56 (42–71)	58 (43–72)	61 (47–74)	< 0.001	58 (42–72)
**Death on or prior to censor date (%)**	226 (74.3)	246 (67.4)	298 (66.2)	289 (53.6)	291 (46)	196 (26.7)	< 0.001	1546 (51.1)
**Bone sarcoma**								
**Number**	77	96	97	94	122	109		595
**Sex**								
Male (%)	46 (59.7)	57 (59.4)	48 (49.5)	52 (55.3)	70 (57.4)	68 (62.4)	0.538	341 (57.3)
Female (%)	31 (40.3)	39 (40.6)	49 (50.5)	42 (44.7)	52 (42.6)	41 (37.6)		254 (42.7)
**Diagnostic age group**								
0–14 years (%)	18 (23.4)	11 (11.5)	17 (17.5)	15 (16)	20 (16.4)	11 (10.1)	0.252	92 (15.5)
15–24 years (%)	15 (19.5)	23 (24)	14 (14.4)	15 (16)	19 (15.6)	27 (24.8)		113 (19)
25–64 years (%)	34 (44.2)	49 (51)	46 (47.4)	43 (45.7)	59 (48.4)	45 (41.3)		276 (46.4)
≥ 65 years (%)	10 (13)	13 (13.5)	20 (20.6)	21 (22.3)	24 (19.7)	26 (23.9)		114 (19.2)
**Median age (IQR)**	29 (16–55)	33.5 (20–55.5)	40 (19–59)	43.5 (19–61)	40.5 (20–63)	42 (19–64)	0.191	38 (18–61)
**Death on or prior to censor date (%)**	44 (57.1)	52 (54.2)	48 (49.5)	53 (56.4)	39 (32)	28 (25.7)	< 0.001	264 (44.4)
**Gastrointestinal stromal sarcoma**^**b**^								
**Number**	0	35		60	67	61		223
**Sex**								
Male (%)	0	18 (51.4)		26 (43.3)	37 (55.2)	38 (62.3)	0.162	119 (53.4)
Female (%)	0	17 (48.6)		34 (56.7)	30 (44.8)	23 (37.7)		104 (46.6)
**Diagnostic age group**								
0–14 years (%)	0	0 0		0	0	0	0.510	0
15–24 years (%)	0	0		31 (52.7)	0	0		114 (51.1)
25–64 years (%)	0	20 (57.1)			37 (55.2)	26 (42.6)		
≥65 years (%)	0	15 (42.9)		29 (48.3)	30 (44.8)	35 (57.4)		109 (48.9)
**Median age (IQR)**	–	56.5 (54–59)	64 (45–78)	64 (53.5–74.5)	65 (50–75)	68 (52–74)	0.931	65 (52–75)
**Death on or prior to censor date (%)**		26 (74.3)		37 (61.7)	36 (53.7)	< 10	< 0.001	107 (48)
**Kaposi’s sarcoma**^**b**^								
**Number**	14	44	31	24	34			147
**Sex**								
Male (%)	11 (78.6)	39 (88.6)	26 (83.9)	20 (83.3)	24 (70.6)		0.303	120 (81.6)
Female (%)	3 (21.4)	5 (11.4)	5 (16.1)	4 (16.7)	10 (29.4)			27 (18.4)
**Diagnostic age group**								
0–14 years (%)	0	0	0	0	0 (0)		0.001	0
15–24 years (%)	< 10	< 10	0	0	13 (38.2)			87 (59.1)
25–64 years (%)	< 10	34 (77.3)	20 (64.5)	10 (41.7)				
≥65 years (%)	< 10	< 10	11 (35.5)	14 (58.3)	21 (61.8)			60 (40.8)
**Median age (IQR)**	58.5 (30–72)	39 (34–60.5)	48 (36–71)	72.5 (52.5–82.5)	75 (62–83)	63 (50–71)	< 0.001	56 (38–75)
**Death on or prior to censor date (%)**	12 (85.7)	40 (90.9)	23 (74.2)	14 (58.3)	10 (29.4)		< 0.001	99 (67.3)
**Total**	395	507	611	717	840	919		3989

*IQR* interquartile range

^a^ Chi-squared or Fisher’s exact test for categorical variables and Kruskal Wallis test for continuous variables

^b^ Less than ten cases denoted as < 10 to protect confidentiality, with some percentage values omitted, sub-periods and/or age groups combined to prevent calculation

### Incidence

**Fig. 1:**
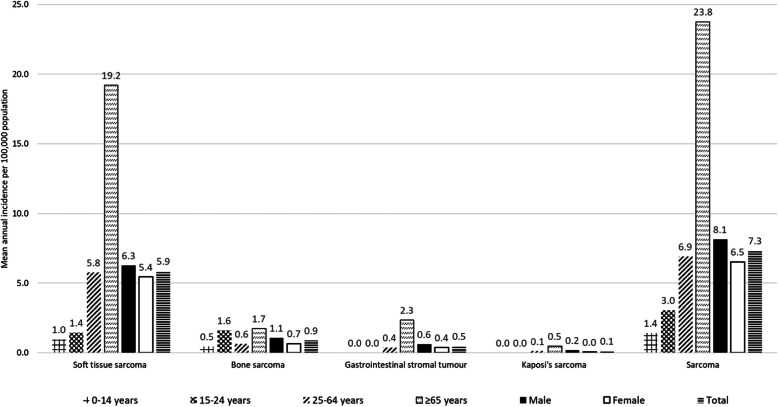
Mean annual crude incidence per 100,000 of sarcoma and major sarcoma groups, by age group and sex for 2012–16

The mean annual age-standardised incidence per 100,000 are provided in Table 2. For sarcoma, there was an increase in incidence over the study period, with the incidence 6.6 per 100,000 for 1982–87 (95% confidence interval (CI) 5.9–7.3), increasing to 9.0 (95% CI 8.4–9.6) for 2012–16. This was underpinned by an increase in STS incidence (5.4 per 100,000 for 1982–87; 7.2 per 100,000 for 2012–16), while there was comparatively little change for bone sarcoma, or Kaposi sarcoma. The limbs were the most common site for STS, with 2.3 per 100,000 in 2012–16, followed by skin (1.4), uterus (0.9) and superficial trunk (0.7). Bone sarcomas were most commonly osteogenic/osteosarcoma (0.3 per 100,000 if 2012–16). Assessing crude incidence for 2012–16 by age group or sex, the highest incidence of STS per 100,000 was for people aged ≥65 years (19.2) and was higher for males (6.3, relative to 5.4 per 100,000 for females) (Fig. 1). Bone sarcoma had an incidence of 1.6 per 100,000 for those aged 15–24 years, which was higher than the 1.4 per 100,000 for STS in this age group and similar to 1.7 per 100,000 for bone sarcoma diagnosed among those aged ≥65 years.

**Table 2:** Mean annual age-standardised^a^ incidence per 100,000 mid-year population (95% confidence interval)

Type of sarcoma^**b**^	1982–87	1988–93	1994–99	2000–05	2006–11	2012–16	Cases
**Soft tissue sarcoma**	5.4 (4.7–6)	5.6 (5–6.2)	6 (5.4–6.6)	5.9 (5.3–6.4)	5.9 (5.5–6.4)	7.2 (6.7–7.8)	3024
Soft tissue sarcoma of head and neck	0.3 (0.1–0.5)	0.2 (0.1–0.3)	0.3 (0.2–0.4)	0.4 (0.3–0.6)	0.2 (0.1–0.3)	0.3 (0.2–0.4)	
Soft tissue sarcoma of limbs	1.2 (0.9–1.5)	1.1 (0.8–1.4)	1.3 (1–1.5)	1.2 (1–1.4)	1.7 (1.4–2)	2.3 (2–2.6)	
Soft tissue sarcoma of superficial trunk	0.3 (0.1–0.4)	0.4 (0.2–0.6)	0.6 (0.4–0.8)	0.6 (0.4–0.8)	0.5 (0.4–0.7)	0.7 (0.6–0.9)	
Soft tissue sarcoma of mediastinum	0.1 (0–0.1)	0 (0–0.1)	0 (0–0.1)	0 (0–0.1)	0 (0–0)	0.1 (0–0.1)	
Soft tissue sarcoma of breast	0.4 (0.2–0.5)	0.1 (0–0.1)	0.2 (0.1–0.4)	0.2 (0.1–0.3)	0.2 (0.1–0.3)	0.3 (0.2–0.4)	
Soft tissue sarcoma of uterus	0.7 (0.4–1)	0.9 (0.6–1.2)	0.9 (0.6–1.2)	1 (0.7–1.3)	0.8 (0.6–1)	0.9 (0.6–1.1)	
Other soft tissue sarcomas of genitourinary tract	0.2 (0.1–0.3)	0.3 (0.2–0.4)	0.2 (0.1–0.3)	0.2 (0.1–0.3)	0.3 (0.2–0.4)	0.1 (0–0.2)	
Soft tissue sarcoma of viscera	0.7 (0.5–0.9)	0.7 (0.5–1)	0.5 (0.3–0.7)	0.3 (0.2–0.4)	0.3 (0.2–0.4)	0.4 (0.2–0.5)	
Soft tissue sarcoma of paratestis	0.2 (0–0.4)	0.2 (0–0.4)	0.2 (0–0.3)	0.1 (0–0.2)	0.1 (0–0.1)	0.1 (0–0.2)	
Soft tissue sarcoma of retroperitoneum and peritoneum	0.4 (0.2–0.6)	0.5 (0.3–0.7)	0.5 (0.3–0.6)	0.3 (0.1–0.4)	0.4 (0.3–0.5)	0.4 (0.3–0.5)	
Soft tissue sarcoma of pelvis	0.2 (0.1–0.4)	0.3 (0.2–0.4)	0.3 (0.1–0.4)	0.2 (0.1–0.3)	0.3 (0.2–0.4)	0.3 (0.2–0.4)	
Soft tissue sarcoma of skin	0.9 (0.6–1.2)	1.1 (0.8–1.3)	1.1 (0.9–1.4)	1.3 (1.1–1.6)	1 (0.8–1.1)	1.4 (1.1–1.6)	
Soft tissue sarcoma of brain and other parts of the nervous system	0.1 (0–0.2)	0.2 (0.1–0.2)	0.1 (0–0.2)	0.1 (0.1–0.2)	0.3 (0.2–0.4)	0.2 (0.1–0.3)	
Embryonal rhabdomyosarcoma of soft tissue	0.1 (0–0.1)	0 (0–0)	0 (0–0)	0.1 (0–0.1)	0.1 (0–0.1)	0 (0–0)	
Ewing’s family tumours of soft tissue	0 (0–0.1)	0 (0–0.1)	0.2 (0.1–0.3)	0.2 (0.1–0.3)	0.1 (0.1–0.2)	0.2 (0.1–0.2)	
**Bone sarcoma**	0.9 (0.7–1.2)	1.1 (0.9–1.3)	1 (0.8–1.3)	0.9 (0.7–1.1)	1 (0.8–1.2)	1 (0.8–1.1)	595
Osteogenic sarcoma	0.3 (0.2–0.5)	0.3 (0.2–0.4)	0.3 (0.2–0.4)	0.3 (0.2–0.3)	0.2 (0.1–0.3)	0.3 (0.2–0.4)	
Chondrogenic sarcoma	0.2 (0.1–0.4)	0.4 (0.2–0.5)	0.3 (0.2–0.4)	0.2 (0.1–0.3)	0.3 (0.2–0.5)	0.2 (0.1–0.3)	
Notochordal sarcomas, chordoma	0.1 (0–0.2)	0.1 (0–0.2)	0.1 (0–0.2)	0.1 (0–0.2)	0 (0–0)	0.1 (0–0.1)	
Ewing’s family of tumours	0.1 (0.1–0.2)	0.1 (0.1–0.2)	0.1 (0–0.2)	0.1 (0.1–0.2)	0.1 (0–0.2)	0.1 (0.1–0.2)	
Other high grade sarcomas (fibrosarcoma, malignant fibrous histiocytoma)	0.1 (0–0.2)	0.1 (0–0.1)	0 (0–0.1)	0 (0–0)	0 (0–0.1)	0 (0–0.1)	
**Gastrointestinal stromal sarcoma**	0 (0–0)	0 (0–0.1)	0.5 (0.3–0.7)	0.7 (0.5–0.9)	0.7 (0.5–0.8)	0.6 (0.5–0.8)	223
**Kaposi’s sarcoma**	0.3 (0.1–0.4)	0.6 (0.4–0.7)	0.4 (0.2–0.5)	0.3 (0.2–0.4)	0.2 (0.1–0.3)	0.1 (0.1–0.2)	147
**Sarcoma (total)**	6.6 (5.9–7.3)	7.3 (6.6–8)	7.9 (7.2–8.5)	7.8 (7.2–8.4)	7.8 (7.2–8.3)	9 (8.4–9.6)	3989
Female breast cancer	115.2 (111–119.4)	130.3 (126.3–134.4)	144.6 (140.7–148.5)	146.1 (142.5–149.6)	146.3 (143–149.5)	162.4 (158.9–165.9)	35,932
Colorectal cancer	84.6 (81.7–87.4)	85.4 (82.9–88)	88.4 (86–90.8)	88.3 (86.1–90.4)	84.4 (82.4–86.3)	71.9 (70.1–73.6)	34,846
Lung Cancer	75.2 (72.5–77.8)	70.8 (68.5–73.1)	69.5 (67.4–71.6)	68.2 (66.3–70.2)	66.2 (64.5–67.9)	63.4 (61.7–65.1)	27,857
Prostate cancer	130.9 (124.6–137.2)	182.3 (176–188.7)	224.8 (218.9–230.7)	214.2 (209.1–219.3)	264.3 (259.3–269.2)	227.6 (223–232.1)	40,802

^a^ Standardised to the 2013 European Standard Population. Note that tier 2 entities do not sum to tier 1 totals

^b^ Soft tissue sarcomas of the heart, paraorbit, alveolar rhabdomyosarcoma, vascular sarcoma and epithelial tumours, adamantinoma all had incidence of zero (to 1 decimal place) throughout and have thus been omitted from the list of sarcoma types

Leiomyosarcoma was the most common STS histology type (17%), followed by malignant fibrous histiocytoma (13%). STS of the head and neck were more often malignant fibrous histiocytoma (21%), while STS of the breast were mainly either Phyllodes tumours (50%) or epitheloid angiosarcomas (22%). STS of the skin had a higher proportion of dermatofibrosarcoma pertuberans (38%) relative to other sites. Bone sarcoma were 23% conventional osteosarcoma, 25% chondrosarcoma and 13% Ewing sarcoma (data not shown).

### Prevalence

At June 2016, there were 1445 prevalent STS cases, 329 bone sarcoma and 48 Kaposi sarcoma. Forty-four percent of prevalent STS cases were diagnosed > 10 years prior, with 19% 5–10 years prior, 26% 1–5 years prior and 11% in the previous year. The corresponding percentages for bone sarcoma were 54, 21, 22 and 4%. STS prevalence increased from 31 to 57 per 100,000 between 1982 and 2016, while bone sarcoma prevalence decreased from 21 per 100,000 to 13 per 100,000 (Additional file 2).

### Relative survival

**Fig. 2:**
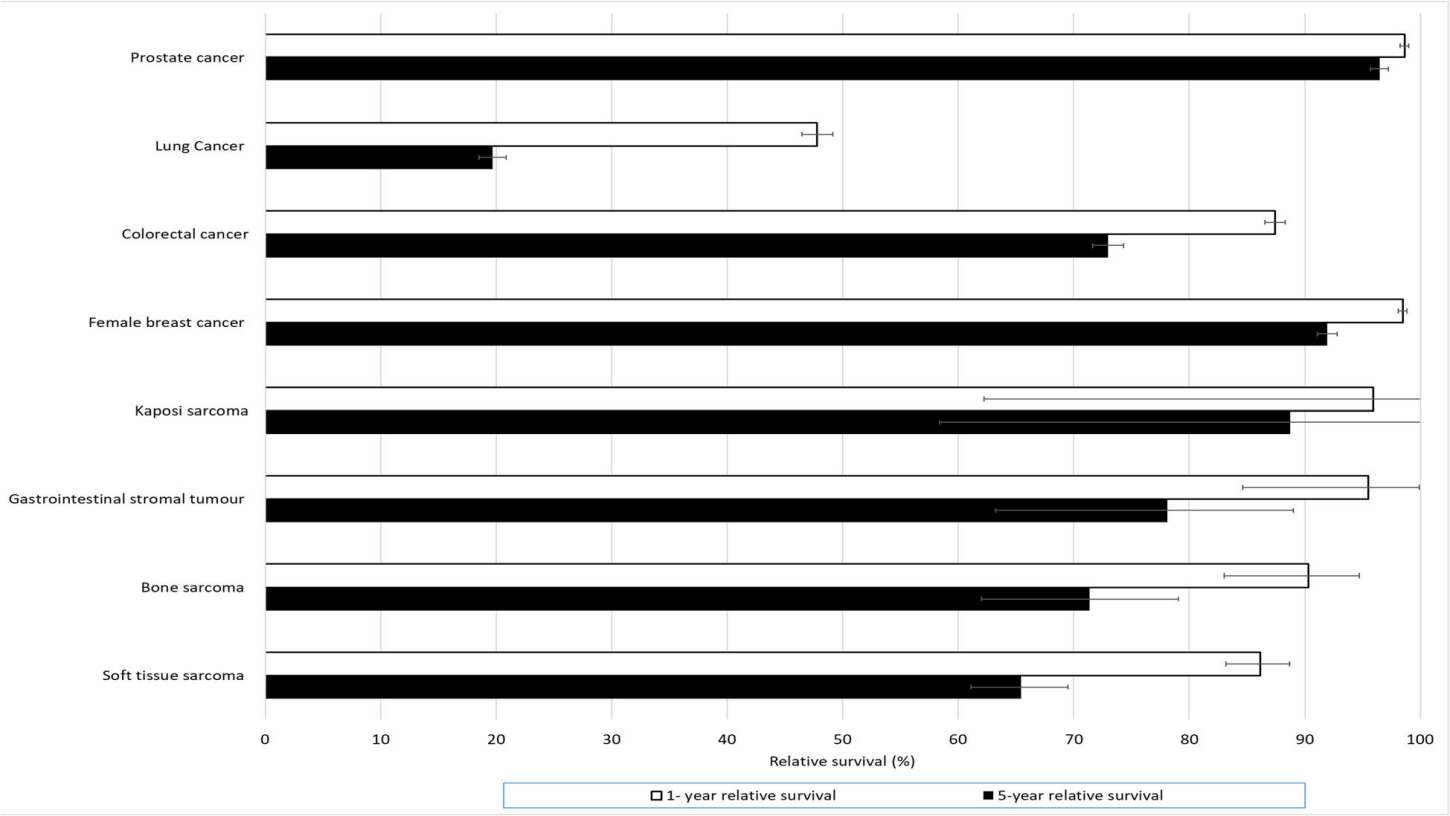
One and five-year relative survival for those ‘at risk’ from 2012 to 16, by cancer type^*^ *Errors bars are 95% confidence intervals

One- and five-year relative survival estimates for tier 1 entities and reference cancers are provided in Fig. 2. Bone sarcoma had a relative survival of 90% at one year and 71% at five years, with the five-year relative of survival of GIST (78%) and Kaposi sarcoma (89%) higher still. One-year relative survival was lower for STS of the viscera (60%), pelvis (66%), brain and other parts of the nervous system (73%) and uterus (78%) relative to other sarcoma types; by contrast STS of the head and neck, limbs and skin had > 90% one-year relative survival. STS of the viscera had a five-year relative survival similar to lung cancer (lung cancer 20%, STS of the viscera 17%), while STS of the breast had a five-year survival almost equal to female breast cancer (~ 91% for both). Relative survival for STS and bone sarcoma stratified by broad age group are provided in Additional file 3.

### Health service use

**Fig. 3:**
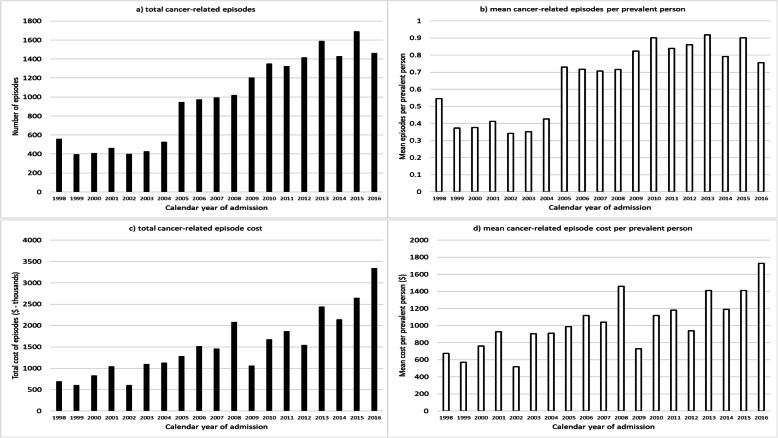
Total episodes of cancer-related hospitalisation (**a**), rate per prevalent person (**b**), total cost (2019) Australian dollars, **c**, and cost per prevalent person (**d**)^*^. *All based on corrected prevalence, described in methods

Cancer-related hospital episodes and costs over the period 1998 to 2016 for sarcoma are shown in Fig. 3. Corresponding figures for the reference cancers are provided in Additional file 4. Over this period there were 18,585 episodes among the cohort with sarcoma, (0.69 episodes per prevalent person) and a total cost of $29.1 million ($3.3 million in 2016). In 1998 the mean cancer-related episodes per prevalent person was 0.54; for 2016 this was 0.76. The associated mean cost per prevalent person increased from $675 to $1728 per prevalent person at risk between 1998 and 2016. The mean cancer-related hospital episodes per prevalent person in 2016 was lower for sarcoma (0.76) than for breast (0.93), colorectal (1.10) and lung cancers (3.23), but higher than for prostate cancer (0.47). Interestingly, the mean cost per prevalent person was the second highest for sarcoma in 2016 ($1728); the reference cancers ranged from $805 (female breast cancer) to $5180 (lung cancer).

## Discussion

To our knowledge, this is the first study of sarcoma epidemiology and health service utilisation in WA, showing an increase in STS from 1982 to 2016. At June 2016, we estimate there were 1935 prevalent cases, with nearly half diagnosed more than 10 years previous.

The definition for sarcoma used in this study was the latest published by RARECARENet [13]. This is a project which collated data from 94 European cancer registries from 27 countries up to 2007. Estimates of age-adjusted incidence are reported for cases diagnosed from 2003 to 2007 (most closely corresponding to the 2000 to 2005 period in this study) [15]. The age-adjusted European incidence of STS was 4.15 per 100,000, lower than 5.9 per 100,000 in WA from 2000 to 2005. The magnitude was also above the (United States (U.S.) standardised) rate of 5.03 per 100,000 reported by Toro et al. [24] using U.S. SEER data from 1978 to 2001. STS of the skin had an incidence in 2000–2005 of 1.3 per 100,000, much higher than 0.30 per 100,000 reported in Europe [15]. The European incidence of bone sarcoma was likewise slightly lower, 0.80 per 100,000 (95% CI 0.78–0.82) compared to 0.9 per 100,000 in WA. The incidence of GIST in WA was markedly higher at 0.7 per 100,000 in WA compared to 0.26 per 100,000 in Europe. The five-year relative survival for STS was higher at 65% than the 57% reported for Europe for 2005–07 [20]; likewise bone sarcoma relative survival was higher (71% compared to 59%, respectively). Because the European data were for an earlier time period and because the ‘relative’ population differed, the above is an indicative rather than ideal comparison.

Australian data for sarcoma produced by the Australian Institute of Health and Welfare are available to 2014 [1], but used a slightly different sarcoma definition (e.g. STS encompasses GIST). The magnitude of incidence is similar (e.g. the age-standardised incidence rate of STS in 2014 was 6 per 100,000, compared to 7.2 per 100,000 in WA). A recent study assessed Australian incidence of STS, standardising to the 2001 Australian population [6]. Replicating this for WA data yields an STS incidence for 2000–2005 of 4.8 per 100,000, compared to 6.2 per 100,000 for Australia. This may be partly explained by a different STS definition used by these authors. Woods et al. [2] in an analysis of sarcoma of the head and neck in Australia from 1982 to 2009 reported an annual incidence rate of 1.59 per 100,000. This is greater than the crude incidence for STS of the head and neck of 0.2 per 100,000 reported for 2012–16 in this study and 0.26 per 100,000 reported by RARECARENet [15]. There were 108 cases of STS of the head and neck in WA reported between 1982 and 2009, according to the classification used in this study. This is much less than 10% (the approximate national proportion of the WA population) of the 3440 cases of skin and STS of the head and neck reported by Woods et al. [2]. The main difference appears to be due to inclusion of STS of the skin and Ewing’s family of tumours in the Woods et al. [2] paper, whereas the RARECARENet classification excludes these from STS of the head and neck and classifies these separately. When considering STS of the skin affecting sites of the face and neck, the number of cases of head and neck sarcoma in WA between 1982 and 2009 increased to 243, closer to 10% of those reported by Woods et al. [2] in their analysis of Australian data.

Bessen et al. [6] also report a similar trend of increasing incidence of STS in Australia between 1982 and 2009, consistent with increases in Europe [15] and the U.S. [24]. The proportionate increase in people aged ≥65 years is consistent with accumulated mutations leading to STS development. Increased vigilance among clinicians, through dissemination of primary care-targeted education material (e.g. [25]) and more clearly defined management guidelines (e.g. [26]) may also have led to more accurate diagnosis of STS over time. Centralised sarcoma management, as occurs through the State Sarcoma Service in WA, could also assist in diagnosis by creating clear referral pathways.

The definition of sarcoma varied in recent studies from China and Germany [27, 28]. Using the RARECARENet definition, this study reported 3989 sarcoma cases. Had the definition by Pingping et al. [27] been used; 4036 cases would have been reported, whereas a broader definition applied by Ressing et al. [28] would have led to 4671 cases being reported in WA. Ressing et al. [28] reported some uncertainty over the inclusion of some morphology codes (e.g. Mullerian mixed tumour), with around 5% removed in these authors' sensitivity analysis. The advantages of using the RARECARENet definition of sarcoma (plus Kaposi sarcoma) is to facilitate comparison of data from WA with those in a different setting. This along with comparison with the four reference cancers contextualises the findings. In Australia, the Australian Comprehensive Cancer Outcomes and Research Database (ACCORD) is a resource currently being utilised for sarcoma epidemiological research [29]. An extension to this study would be to compare ACCORD estimates with those in the WACR.

Having access to health service utilisation information provides an additional lens to this epidemiological analysis. Cancer-related hospitalisation related to cancer generally, rather than sarcoma specifically. Anecdotal feedback from the State Sarcoma Service indicated that many referrals and effort in management are directed towards benign or borderline lesions, as even if without metastatic potential these can be locally aggressive and difficult to manage. While those included on the WACR have been mentioned in the results as specific exclusions, sarcoma is by definition a malignant disease and thus is would be inappropriate to include these lesions in the analysis. Finally, the prevalence analysis cannot account for inward and outward inter-state or international migration post-diagnosis.

## Conclusions

Because sarcoma is a rare cancer, understanding epidemiological trends and health resource utilisation is important to planning centralised management of patients through a multi-disciplinary team with sarcoma expertise. This study adds to a relatively small pool of literature analysing sarcoma epidemiology, especially in Australia. STS incidence has increased over time in WA, with an increasing proportion of people diagnosed aged ≥65 years. Bone sarcoma remains rare, but younger people make up a higher proportion of the total case load, compared to other forms of sarcoma. Management can have long-term quality of life implications (e.g. if limb amputation is indicated) and for a proportion of people the survival outcome is poor. The analysis of health service use showed sarcoma had a lower mean episode per prevalent person of cancer-related hospitalisation compared to the reference cancers in 2016, but the mean cost per prevalent person was higher for sarcoma than for female breast, colorectal and prostate cancer.

## Supplementary information

**Additional file 1.** Sarcoma topographical and morphological codes and codes for cancer-related hospitalisations.

**Additional file 2.** Corrected prevalence by year, 1982 to 2016.

**Additional file 3.** One- and five-year relative survival for soft tissue sarcoma and bone sarcoma, stratified by age group.

**Additional file 4.** Total episodes of cancer-related hospitalisation (a), rate per prevalent person (b), total associated cost (2019) Australian dollars, c, and cost per prevalent person based on corrected prevalence (d).

## Data Availability

The datasets generated and/or analysed during the current study are not publicly available due to an ethics and research governance process being in place to obtain the data used.

## Abbreviations

ABS: Australian Bureau of Statistics
GIST: Gastrointestinal stromal tumour
HMDC: Hospital morbidity data collection
ICD: International Classification of Disease
RARECARENet: Information Network on Rare Cancers
RECORD: Reporting of studies Conducted using Observational Routinely-collected health Data
STS: Soft tissue sarcoma
U.S: United States
WA: Western Australia
WACR: WA Cancer Registry

## References

[1] Cancer Australia (2018). Sarcoma statistics Canberra.

[2] Woods RH, Potter JA, Reid JL, Louise J, Bessen T, Farshid G (2018). Patterns of head and neck sarcoma in Australia. ANZ J Surg.

[3] Youlden DR, Aitken JF (2019). Childhood cancer in Australia, 1983–2015.

[4] Blackwell JB, Threlfall TJ, McCaul KA (2005). Primary malignant bone tumours in Western Australia, 1972-1996. Pathology.

[5] Gibson E, Woods RH, Potter JA, et al. Epidemiological trends in dermal sarcoma in Australia. Australas J Plast Surg. 2019;2(2):10–6.

[6] Bessen T, Caughey GE, Shakib S, Potter JA, Reid J, Farshid G (2019). A population-based study of soft tissue sarcoma incidence and survival in Australia: an analysis of 26,970 cases. Cancer Epidemiol.

[7] Australian Government (2019). Department of Health. Clinical Trial Activity: Rare Cancers and Rare Diseases and Unmet Needs.

[8] Benchimol EI, Smeeth L, Guttmann A, Harron K, Moher D, Petersen I (2015). The REporting of studies conducted using observational routinely-collected health data (RECORD) statement. PLoS Med.

[9] Holman CD, Bass AJ, Rouse IL, Hobbs MS (1999). Population-based linkage of health records in Western Australia: development of a health services research linked database. Aust N Z J Public Health.

[10] Australian Bureau of Statistics. 3101.0. Australian Demographic Statistics, Sep 2018. Table 4. Estimated Resident Population, States and Territories (Number). 2018. https://www.abs.gov.au/AUSSTATS/abs@.nsf/DetailsPage/3101.0Sep%202018?OpenDocument. Accessed 24 Apr 2019.

[11] International Association of Cancer Registries (2004). International rules for multiple primary cancers.

[12] Australian Institute of Health and Welfare (2012). Cancer survival and prevalence in Australia: period estimates from 1982 to 2010. Cancer Series no. 69. Cat. no. CAN 65.

[13] Information Network on Rare Cancers (2015). List of rare cancers.

[14] Gatta G, Capocaccia R, Botta L (2017). Burden and centralised treatment in Europe of rare tumours: results of RARECAREnet-a population-based study. Lancet Oncol.

[15] Information Network on Rare Cancers (2020). Age-adjusted incidence over time in Europe.

[16] Maxwell S, O'Leary P, Slevin T (2014). The increase in cancer prevalence and hospital burden in Western Australia, 1992-2011. Popul Health Metrics.

[17] Ederer FH, H. (1959). Methodological note no. 10, end results evaluation section. Instructions to IBM 650 programmers in processing survival computations.

[18] Brenner H, Soderman B, Hakulinen T (2002). Use of period analysis for providing more up-to-date estimates of long-term survival rates: empirical evaluation among 370,000 cancer patients in Finland. Int J Epidemiol.

[19] Baade P, Cramb S, Dasgupta P, Youlden D (2016). Estimating cancer survival - improving accuracy and relevance. Aust N Z J Public Health.

[20] Information Network on Rare Cancers (2020). 1, 3 and 5-year Relative Survival (RS) overtime in Europe.

[21] Australian Bureau of Statistics (2016). Deaths, Year of registration, Age at death, Age-specific death rates, Sex, States, Territories and Australia.

[22] Independent Hospitals Pricing Authority (2020). National Efficient Price Determination.

[23] Australian Bureau of Statistics (2019). Tables 1 and 2. CPI: All Groups, Index Numbers and Percentage Changes.

[24] Toro JR, Travis LB, Wu HJ, Zhu K, Fletcher CD, Devesa SS (2006). Incidence patterns of soft tissue sarcomas, regardless of primary site, in the surveillance, epidemiology and end results program, 1978-2001: an analysis of 26,758 cases. Int J Cancer.

[25] Pike J, Clarkson PW, Masri BA (2008). Soft tissue sarcomas of the extremities: how to stay out of trouble. B C Med.

[26] Dangoor A, Seddon B, Gerrand C, Grimer R, Whelan J, Judson I (2016). UK guidelines for the management of soft tissue sarcomas. Clin Sarcoma Res.

[27] Pingping B, Yuhong Z, Weiqi L, Chunxiao W, Chunfang W, Yuanjue S (2019). Incidence and mortality of sarcomas in Shanghai, China, during 2002-2014. Front Oncol.

[28] Ressing M, Wardelmann E, Hohenberger P, Jakob J, Kasper B, Emrich K (2018). Strengthening health data on a rare and heterogeneous disease: sarcoma incidence and histological subtypes in Germany. BMC Public Health.

[29] Australian and New Zealand Sarcoma Association (2020). Clinical Trials and Studies: Advanced Soft Tissue Sarcoma Study.

