# Supplementary material for: Sarcoma epidemiology and cancer-related hospitalisation in Western Australia from 1982 to 2016: a descriptive study using linked administrative data
Source: BMC Cancer. 2020 Jul 6;20:625. doi: 10.1186/s12885-020-07103-w (PMC7336405; doi:10.1186/s12885-020-07103-w)
Supplement: Supplementary file 1 — Additional file 1. Sarcoma topographical and morphological codes and codes for cancer-related hospitalisations. [file 12885_2020_7103_MOESM1_ESM.docx]

Additional file 1. Sarcoma topographical and morphological codes and codes for cancer-related hospitalisations.

Table 1. Classification of sarcoma (using Information Network on Rare Cancers (RARECARENet) classifications (13).

| **Tumour** | **ICD-O-3 Topography code** | **ICD-O-3 Morphology code** |
| --- | --- | --- |
| Soft tissue sarcoma | all cancers sites except C40.0-C41.9 | **all cancers sites except C40.0-C41.9 (includes unknown primary sites) :** 8710-8711, 8800-8935, 8959, 8963-8964, 8990-8991, 9020, 9040-9044, 9120-9133, 9150, 9170, 9180, 9231, 9240, 9251, 9260,9364-9365, 9540, 9560-9571, 9580-9581 **all cancer sites except C40.0-C41.9,C7-C8,C60,C44,C63.2:** 8940 **all cancer sites except C40.0-C41.9,C56, C62, C71, C72**: 9473 |
| Bone sarcoma | C40.0-C41.9 | 8800-8801, 8803-8806, 8810, 8811-8812, 8815, 8830, 8840, 8850-8855, 8890-8891, 8894-8896, 8900-8902, 8910, 8912, 8920, 9040-9044, 9120-9133, 9150, 9170, 9180-9250, 9260-9261, 9310, 9364, 9370-9372, 9540-9581 |
| Gastrointestinal stromal tumour | any sites | 8936 |
| Kaposi's sarcoma | any sites | 9140 |

ICD-O = International Classification of Disease – Oncology. For tier 2 and 3 classifications, see reference.

Table 2. Diagnostic and procedure codes for cancer-related hospitalisations.

| **Code type** | **Codes** |
| --- | --- |
| International Classification of Disease – 9 – Canadian Modification (Australian Version 2) | 140.00 – 208.99; 235.00-239.99 |
| International Classification of Disease – 10 – Australian Modification | C00.0 – C96.99; D37.0 – D48.9 |
| Procedure codes^a^ | 96196-00, 96197-00, 96198-00, 96199-00, 96200-00, 96201-00, 96202-00, 96203-00, 96204-00, 96205-00, 96206-00, 96207-00, 96208-00, 96209-00, 90760-00 - 90762-00, 99.25, 6-310 – 6-319, 6-215 – 6-222, 63.10 – 63.19, 62.15- 62.22, 15000-00 – 15600-04, 15303-00 – 15367-00, 15012-00 – 15012-01, 90766-00, 90764-00, 90764-01, 16000-00, 16012-00 – 16015-00, 15342-00 – 15351-00, 90765-00 – 90765-04, 92.20 – 92.29, 3-060, 3-096, 3-097, 3-700 – 3-799, 3-800 – 3-899, 30.60, 30.96, 30.97, 37.00 – 37.99, 38.00 – 38.99, 30600-00, 30960-00, 30970-00, 37000-00 – 37999-99, 38000-00 – 38999-99 |

1. Relevant codes for study period are from International Classification of Disease – 9 – Canadian Modification (Australian Version 2) and the Australian Classification of Health Interventions 1^st^ – 7^th^ editions.
