# Supplementary figures and images for: Sarcoma epidemiology and cancer-related hospitalisation in Western Australia from 1982 to 2016: a descriptive study using linked administrative data
Source: BMC Cancer. 2020 Jul 6;20:625. doi: 10.1186/s12885-020-07103-w (PMC7336405; doi:10.1186/s12885-020-07103-w)

Additional file 2. Corrected prevalence by year, 1982 to 2016 (note different scales on y-axis)


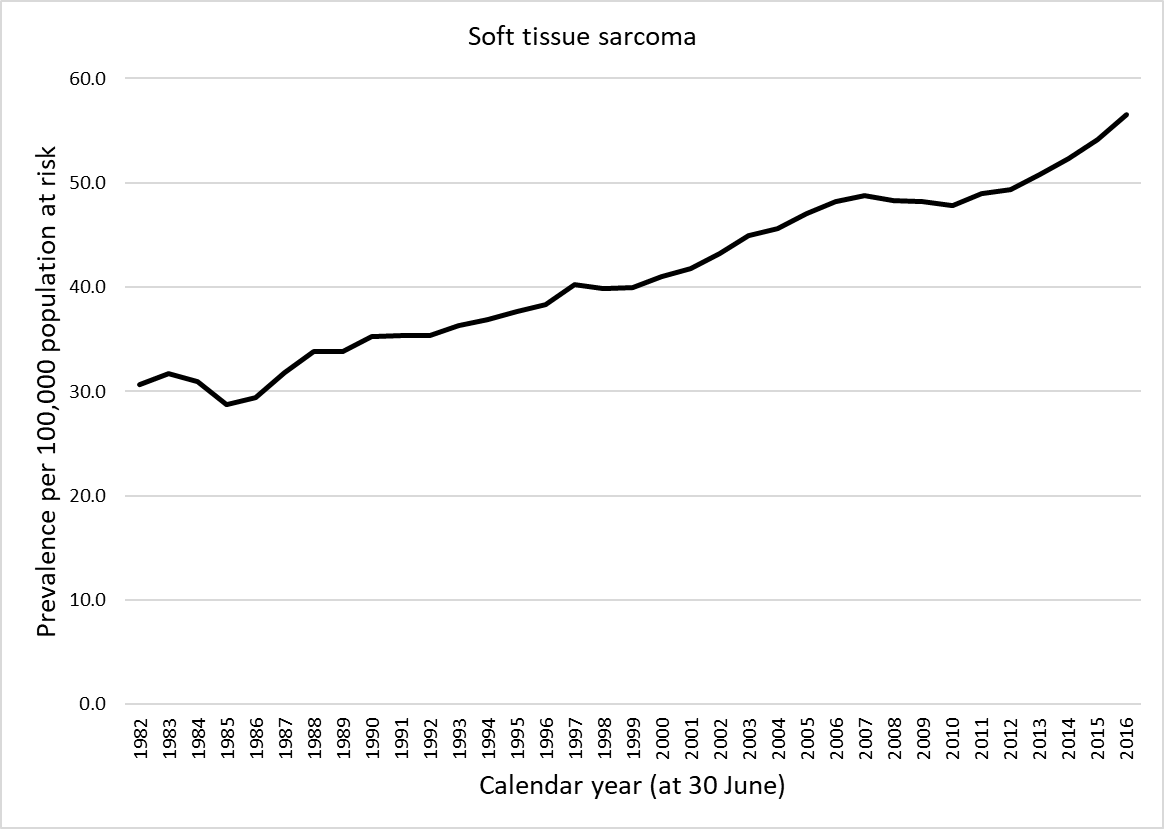


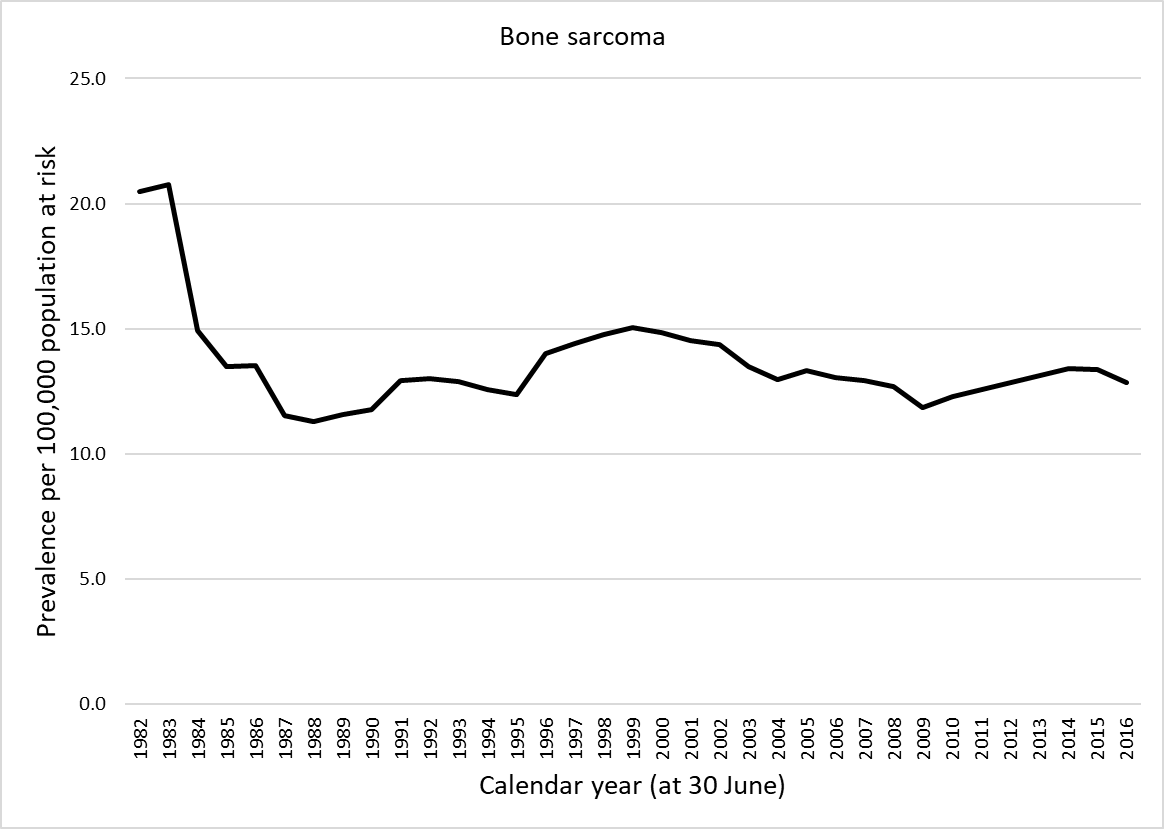


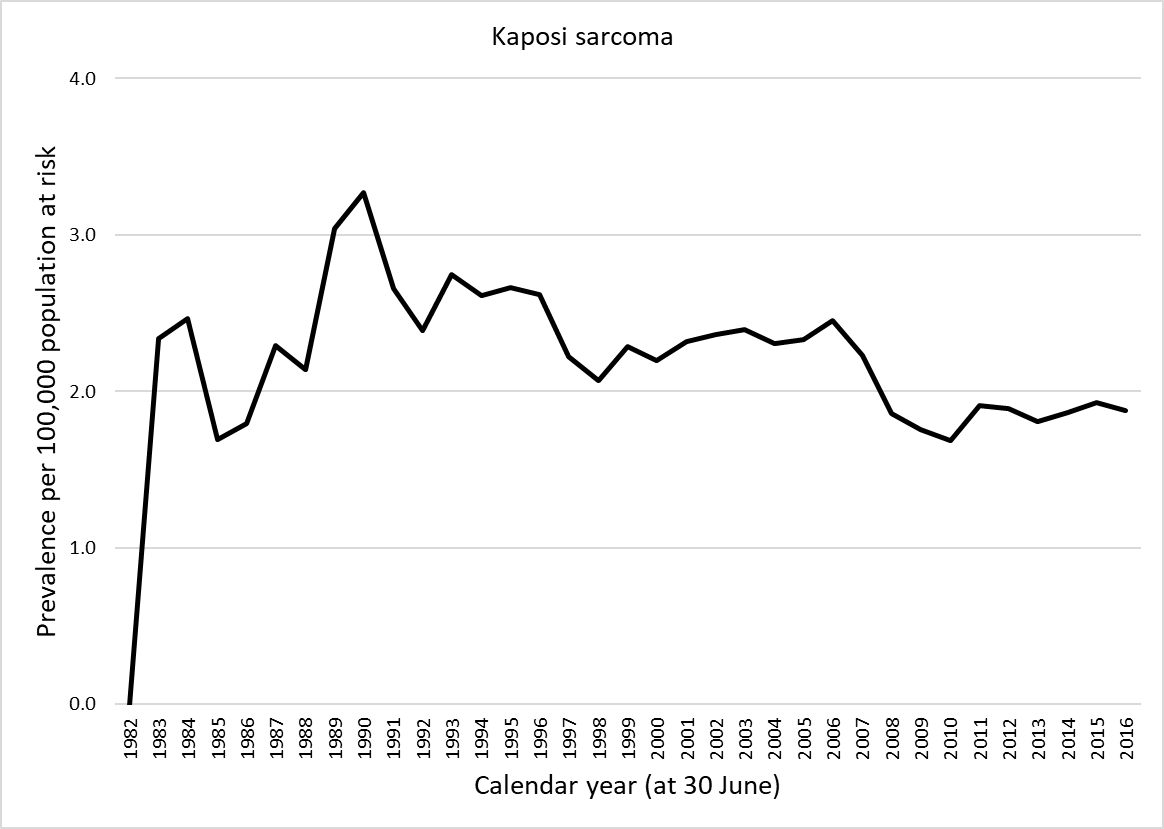

Supplement: Supplementary file 2 — Additional file 2. Corrected prevalence by year, 1982 to 2016. [file 12885_2020_7103_MOESM2_ESM.docx]
