# Supplementary material for: Sarcoma epidemiology and cancer-related hospitalisation in Western Australia from 1982 to 2016: a descriptive study using linked administrative data
Source: BMC Cancer. 2020 Jul 6;20:625. doi: 10.1186/s12885-020-07103-w (PMC7336405; doi:10.1186/s12885-020-07103-w)

Additional file 3. One- and five-year relative survival for soft tissue sarcoma and bone sarcoma, stratified by age group. Error bars are 95% confidence intervals.


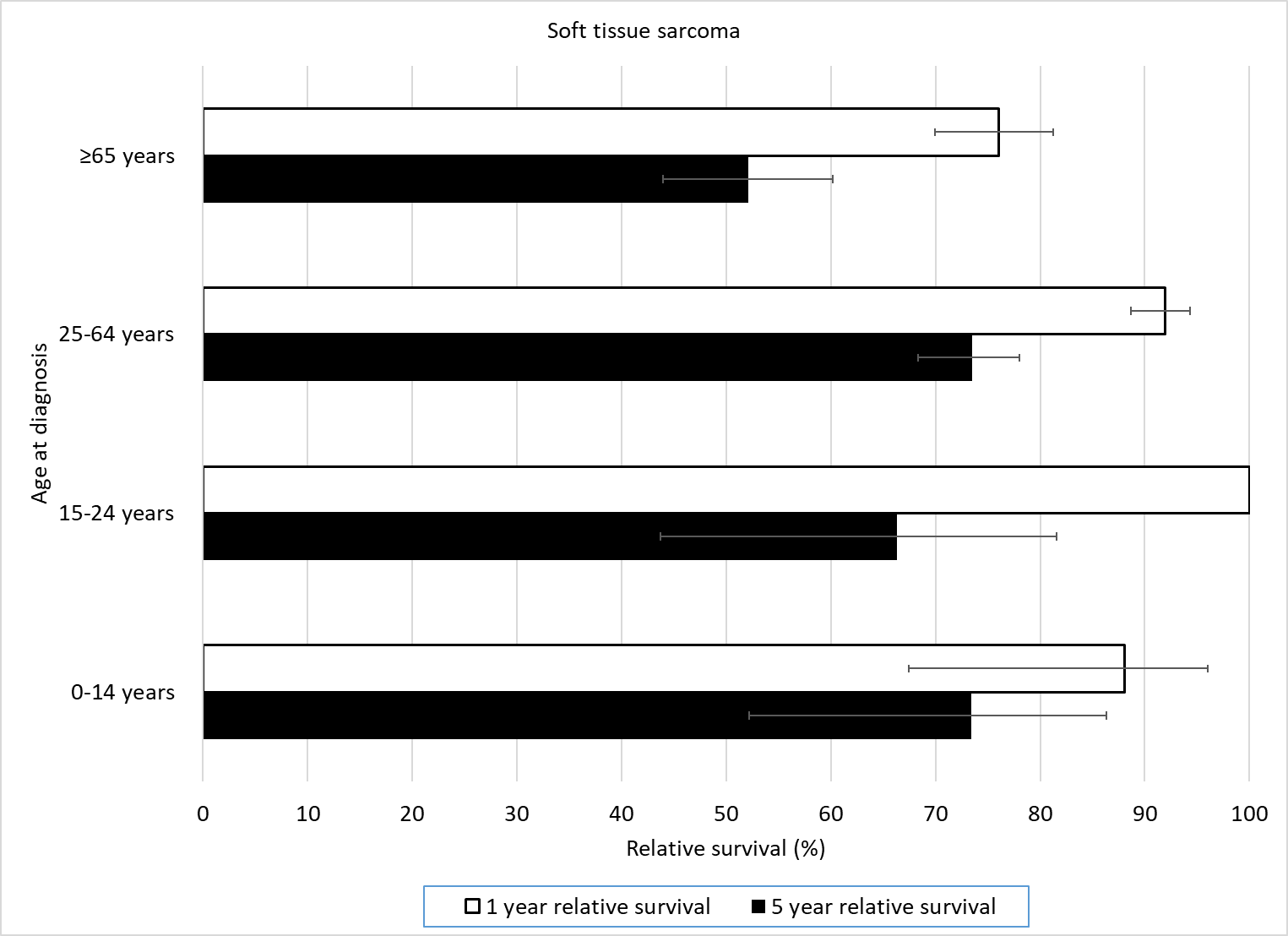


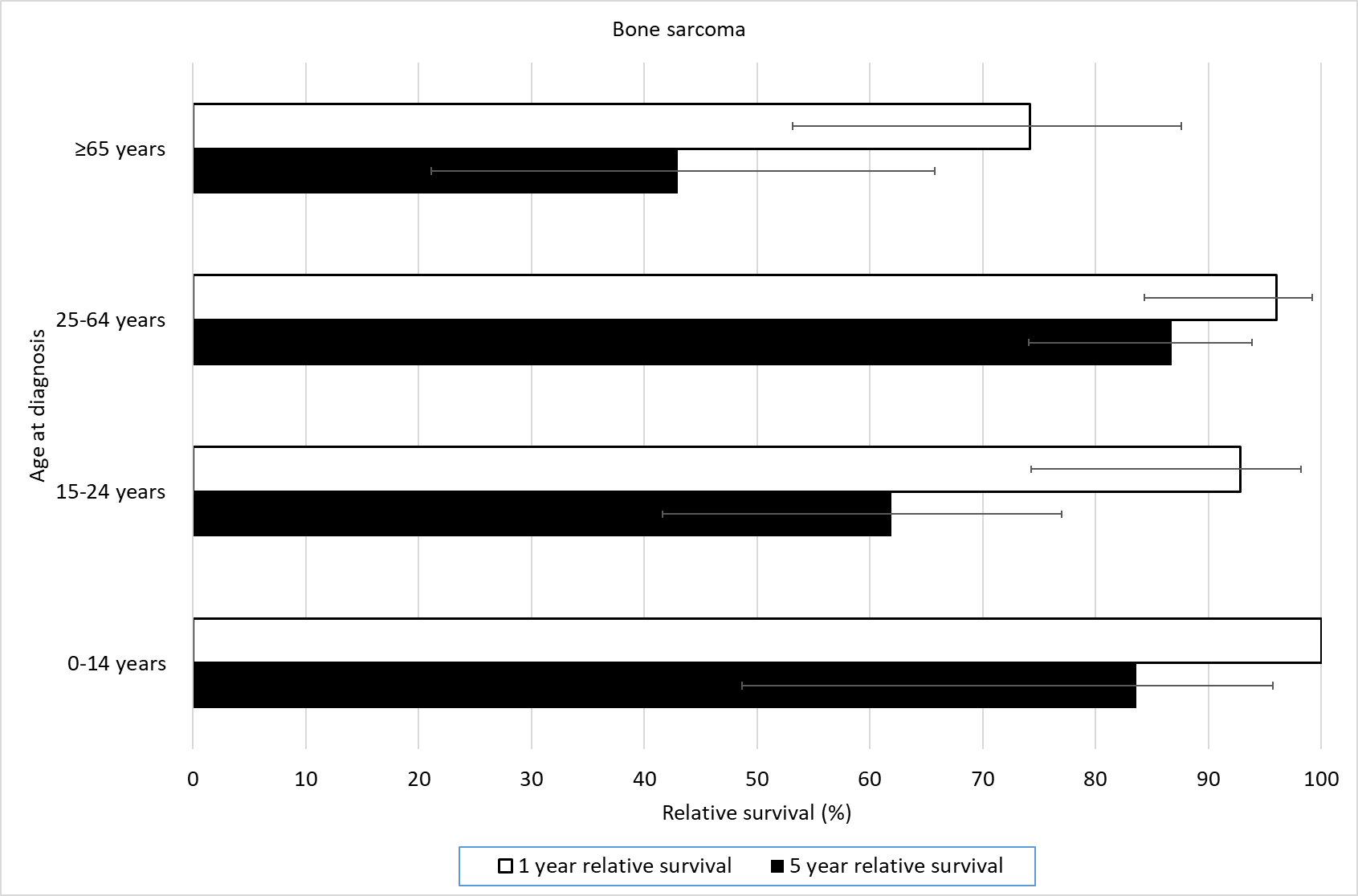

Supplement: Supplementary file 3 — Additional file 3. One- and five-year relative survival for soft tissue sarcoma and bone sarcoma, stratified by age group. [file 12885_2020_7103_MOESM3_ESM.docx]
