# Supplementary material for: Sarcoma epidemiology and cancer-related hospitalisation in Western Australia from 1982 to 2016: a descriptive study using linked administrative data
Source: BMC Cancer. 2020 Jul 6;20:625. doi: 10.1186/s12885-020-07103-w (PMC7336405; doi:10.1186/s12885-020-07103-w)

Additional file 4. Total episodes of cancer-related hospitalisation (a), rate per prevalent person (b), total associated cost (2019 Australian dollars, c), and cost per prevalent person (d) based on corrected prevalence.

1. Female breast cancer


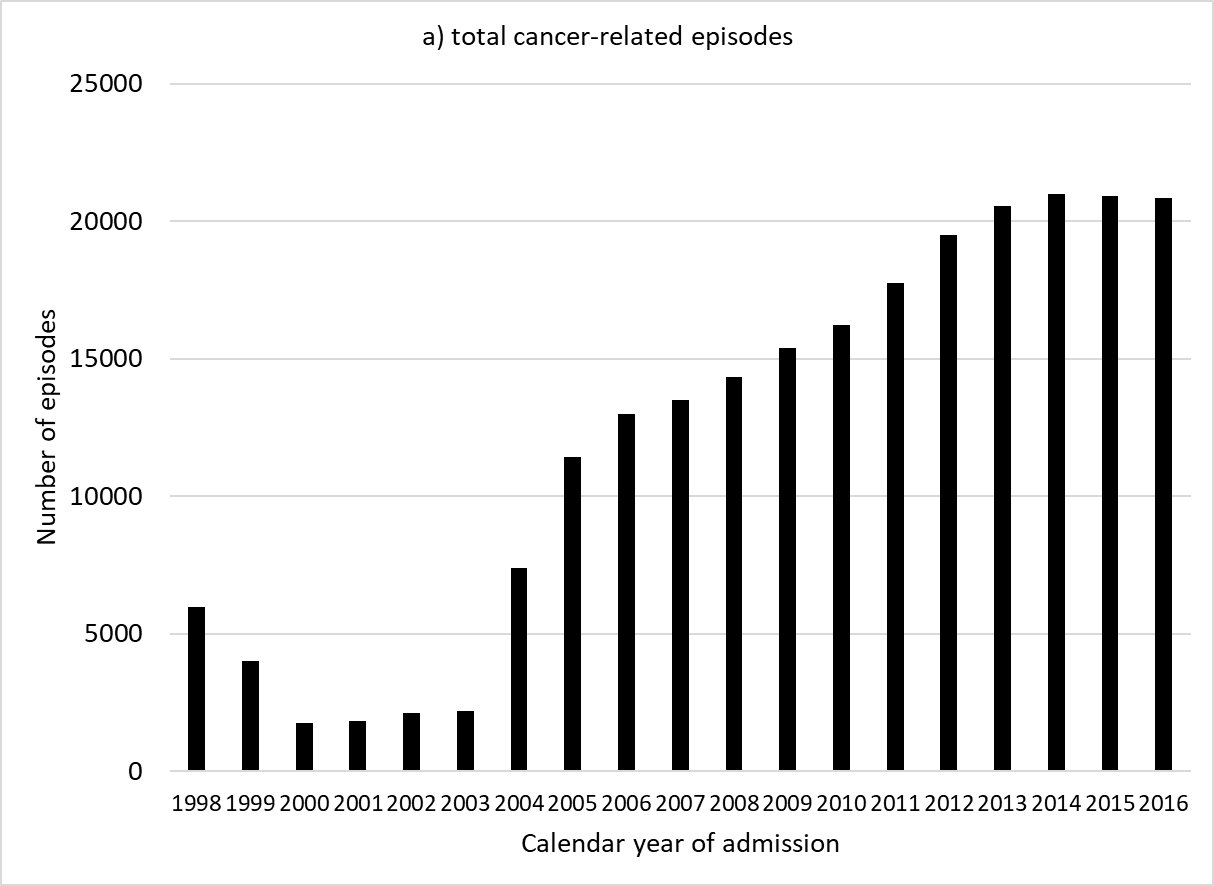

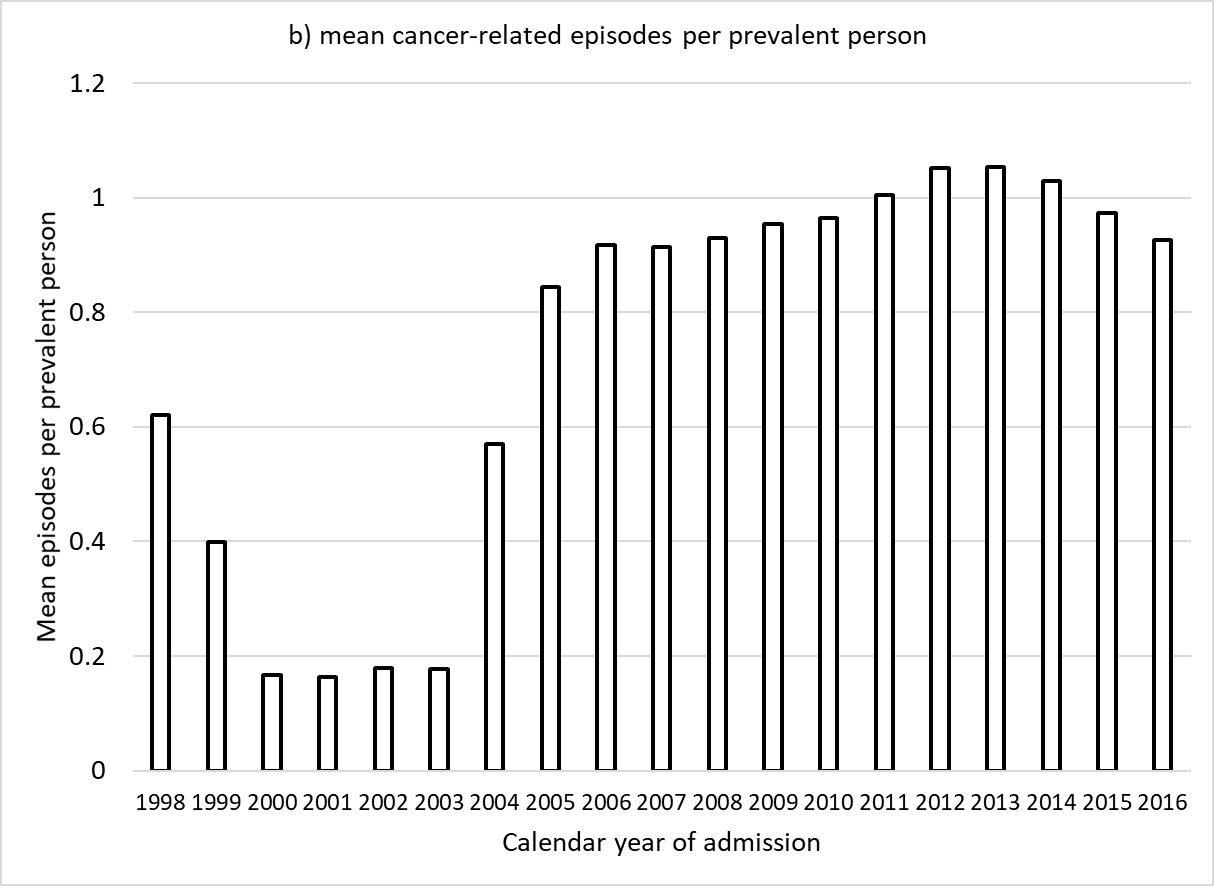

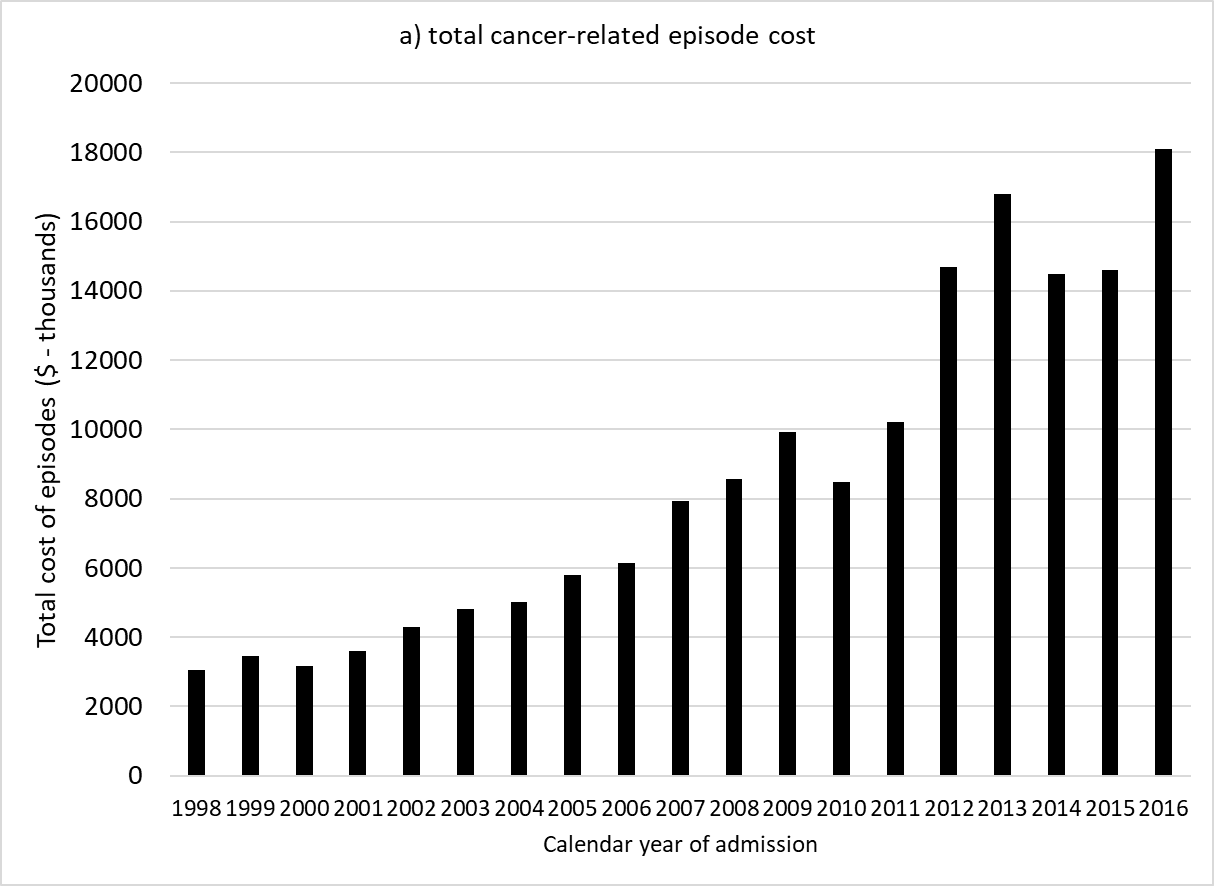

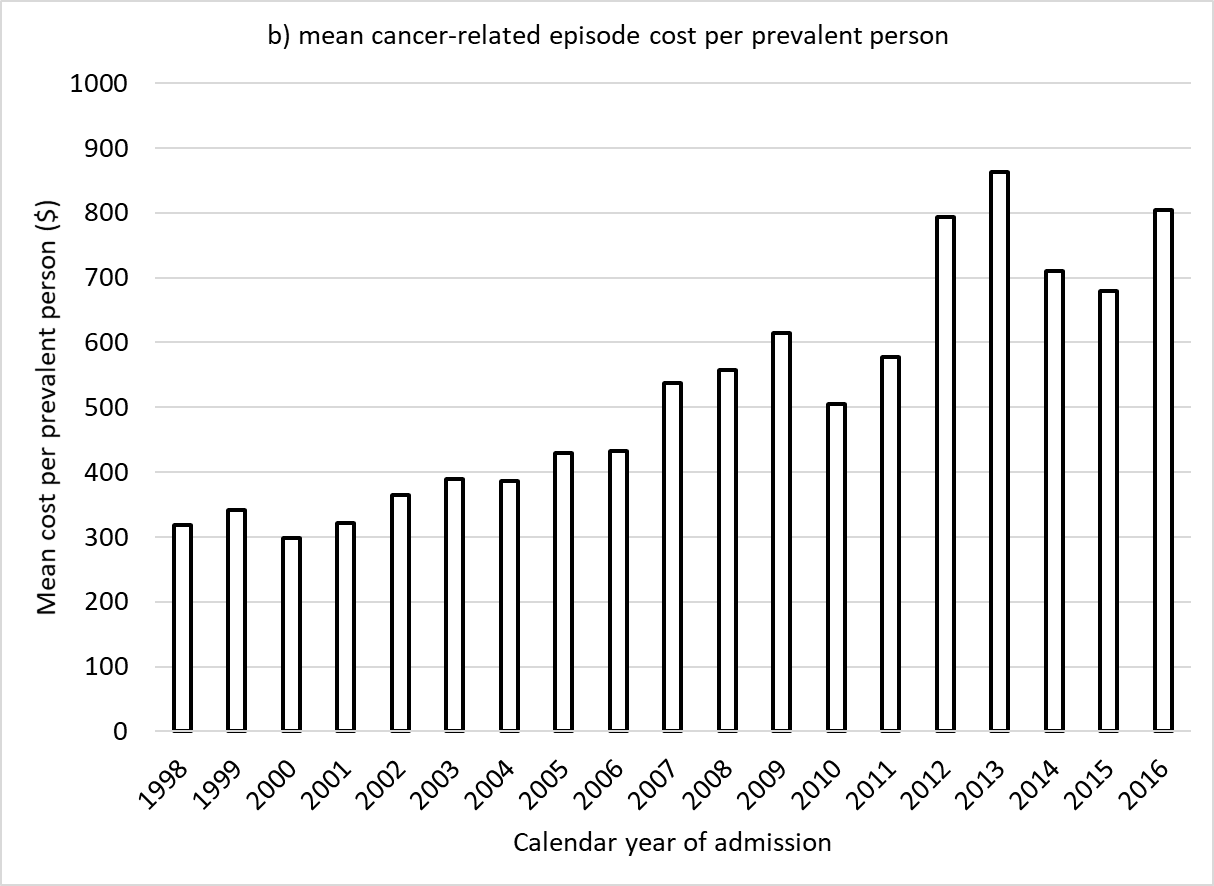


1. Colorectal cancer


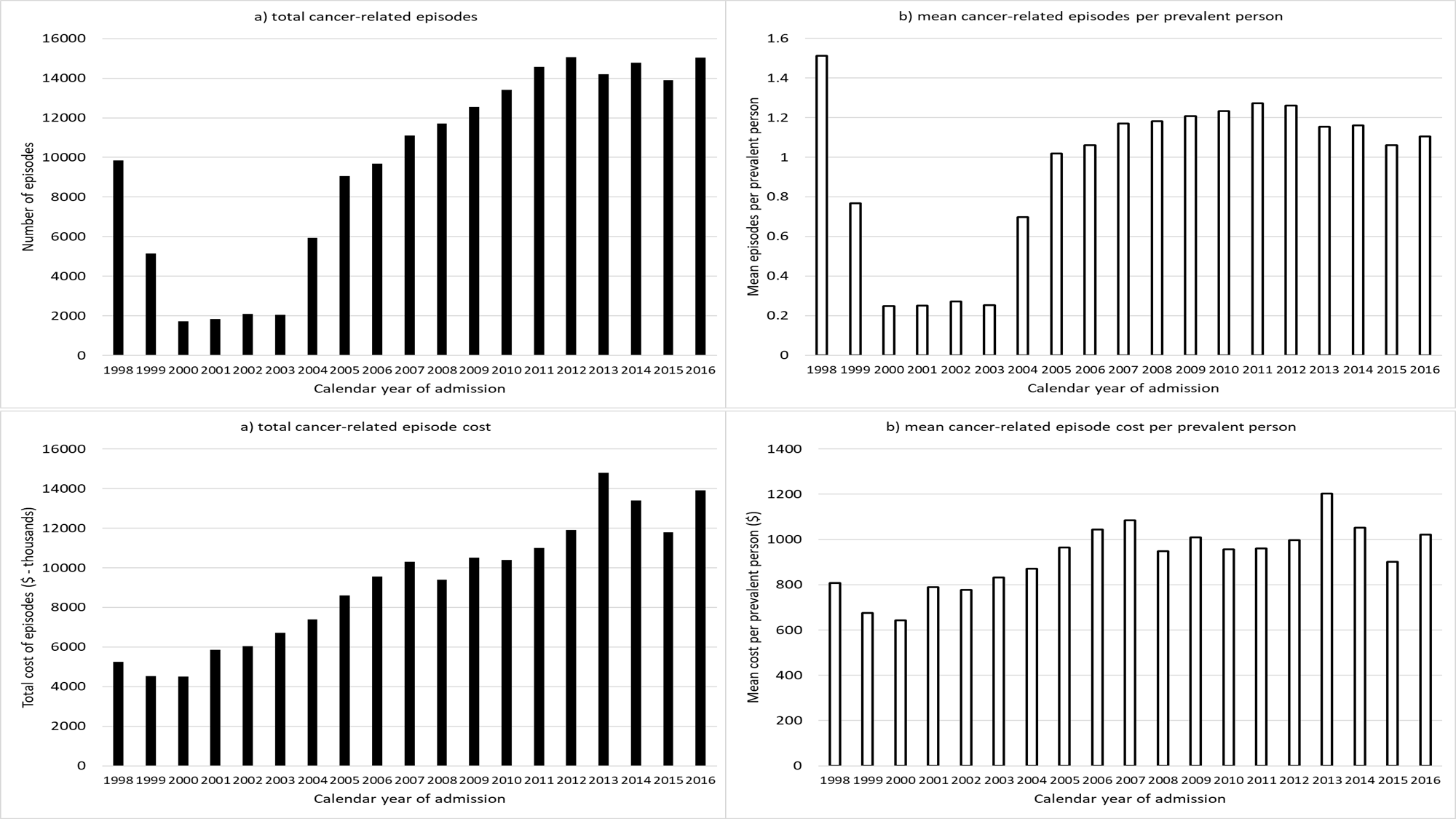


1. Lung cancer


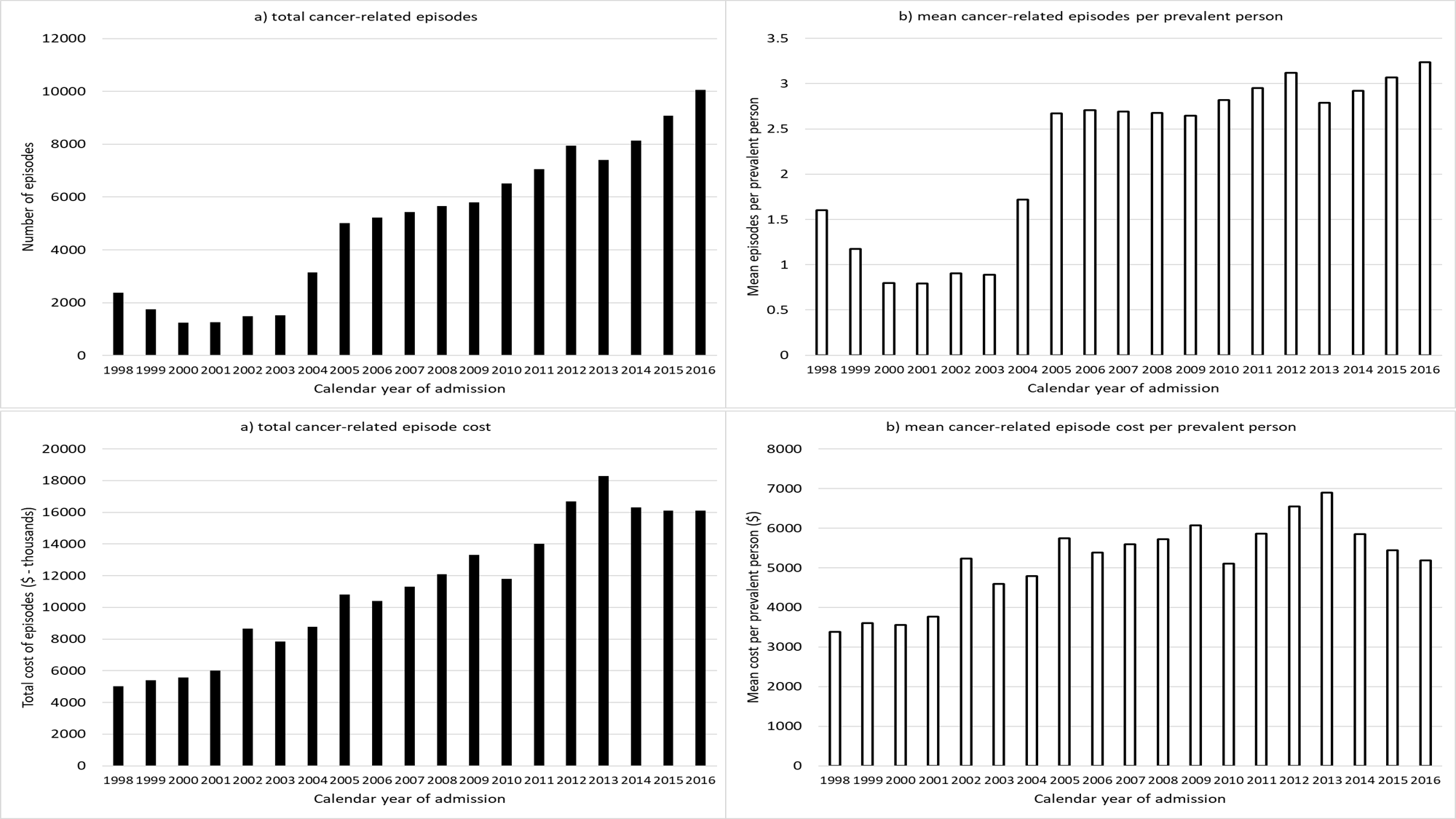


1. Prostate cancer


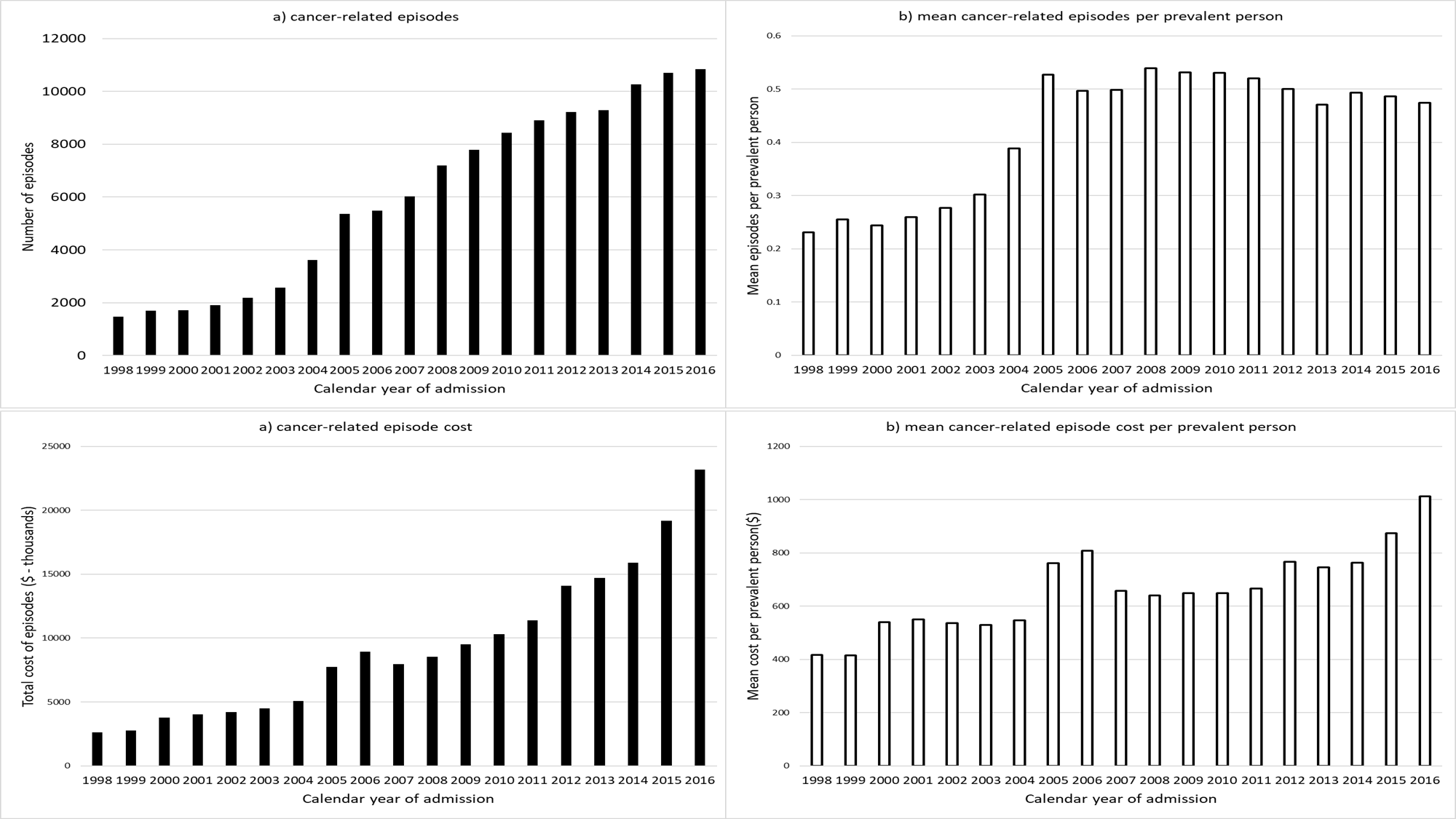

Supplement: Supplementary file 4 — Additional file 4. Total episodes of cancer-related hospitalisation (a), rate per prevalent person (b), total associated cost (2019) Australian dollars, c, and cost per prevalent person based on corrected prevalence (d). [file 12885_2020_7103_MOESM4_ESM.docx]
